# Insight into the Binding of Argon to Cyclic Water Clusters from Symmetry-Adapted Perturbation Theory

**DOI:** 10.3390/ijms242417480

**Published:** 2023-12-14

**Authors:** Carly A. Rock, Gregory S. Tschumper

**Affiliations:** Department of Chemistry and Biochemistry, University of Mississippi, University, MS 38677-1848, USA

**Keywords:** water clusters, argon tagging, vibrational frequencies, interaction energies, binding energies

## Abstract

This work systematically examines the interactions between a single argon atom and the edges and faces of cyclic H${}_{2}$O clusters containing three–five water molecules (Ar(H${}_{2}$O)${}_{n=3–5}$). Full geometry optimizations and subsequent harmonic vibrational frequency computations were performed using MP2 with a triple-ζ correlation consistent basis set augmented with diffuse functions on the heavy atoms (cc-pVTZ for H and aug-cc-pVTZ for O and Ar; denoted as haTZ). Optimized structures and harmonic vibrational frequencies were also obtained with the two-body–many-body (2b:Mb) and three-body–many-body (3b:Mb) techniques; here, high-level CCSD(T) computations capture up through the two-body or three-body contributions from the many-body expansion, respectively, while less demanding MP2 computations recover all higher-order contributions. Five unique stationary points have been identified in which Ar binds to the cyclic water trimer, along with four for (H${}_{2}$O)${}_{4}$ and three for (H${}_{2}$O)${}_{5}$. To the best of our knowledge, eleven of these twelve structures have been characterized here for the first time. Ar consistently binds more strongly to the faces than the edges of the cyclic (H${}_{2}$O)${}_{n}$ clusters, by as much as a factor of two. The 3b:Mb electronic energies computed with the haTZ basis set indicate that Ar binds to the faces of the water clusters by at least 3 kJ mol${}^{-1}$ and by nearly 6 kJ mol${}^{-1}$ for one Ar(H${}_{2}$O)${}_{5}$ complex. An analysis of the interaction energies for the different binding motifs based on symmetry-adapted perturbation theory (SAPT) indicates that dispersion interactions are primarily responsible for the observed trends. The binding of a single Ar atom to a face of these cyclic water clusters can induce perturbations to the harmonic vibrational frequencies on the order of 5 cm${}^{-1}$ for some hydrogen-bonded OH stretching frequencies.

## 1. Introduction

Noble gases are frequently used as carrier gases and as inert environments in a broad range of spectroscopic techniques. Supersonic expansions [1,2,3,4,5,6,7,8,9,10,11,12], cryogenic matrices [13,14,15,16,17,18,19,20,21,22,23,24,25,26,27,28,29,30,31,32,33,34,35], and helium nanodroplets [36,37,38,39,40,41,42] are specific examples that continue to play important roles in the experimental characterization of weakly bound molecular complexes. Spectroscopic studies of neutral hydrogen-bonded clusters, including H${}_{2}$O clusters, sometimes utilize one or more noble gas atoms (typically Ar) as an experimental tag to probe structural features, enhance experimental signals and even examine the hydrophobic effect [43,44,45,46,47,48,49,50,51,52,53,54,55,56,57,58,59,60,61,62,63,64,65,66,67,68,69,70,71].

Although the noble gases are inert under these types of experimental conditions, they can still perturb the molecules and complexes being studied [3,7,31,51,55,72,73,74,75,76,77]. Several studies involving Ar-tagged complexes demonstrate the potential of Ar to engage in favorable intermolecular dispersion and induction interactions when utilized as an isotropic probe of electron density to provide insight into regions of a molecule or molecular cluster of interest [51,53,54,58,59,62,63,64,65,68,78].

The interaction between an Ar atom and a single H${}_{2}$O molecule has been studied in great detail [69,79,80,81,82,83,84,85,86,87,88,89,90,91,92,93], but relatively few studies have looked at the interactions of Ar with the water dimer [69,87,94] or larger water clusters [95,96]. The present study systematically identifies the energetically favorable binding sites of a single Ar atom to the well-characterized structures of cyclic (H${}_{2}$O)${}_{n=3–5}$ clusters [6,10,24,30,31,37,42,97,98,99,100,101,102,103,104,105,106,107,108,109,110,111,112,113,114,115,116,117,118,119,120,121,122,123,124,125,126,127,128,129,130,131,132,133,134,135,136] while also tracking structural and vibrational perturbations that occur. Symmetry-adapted perturbation theory is used to analyze the interaction energies for the different binding motifs.

## 2. Computational Details

The lowest-energy binding sites of a single Ar atom around small water clusters with three–five water molecules (Ar(H${}_{2}$O)${}_{n=3–5}$) were identified via full geometry optimizations and harmonic vibrational frequency computations using MP2 [137] and Dunning’s correlation-consistent cc-pVTZ [138] basis set for H atoms and aug-cc-pVTZ [139,140] for the “heavy atoms” (O and Ar), hereafter denoted as haTZ. A subsequent set of haTZ geometry optimizations and harmonic vibrational frequency computations were performed on the MP2/haTZ-identified stationary points with the highly efficient and accurate *N*-body–many-body (*N*b:Mb) technique [134,141,142,143] that captures all leading dominant *N*-body contributions to the many-body expansion (MBE) of the interactions in a cluster using an accurate high-level method, whereas the remaining higher-order contributions are recovered with a less demanding low-level method. For this study, we have selected the 2b:Mb [142,143,144,145,146,147] and 3b:Mb [141,143] versions of the *N*b:Mb procedure. In this implementation, CCSD(T) [148] is used as the high-level method to describe the one- and two-body terms in the MBE of the cluster energy for 2b:Mb (as well as the three-body interactions for 3b:Mb) while MP2 is used as the low-level method to recover the higher-order ≥ three-body contributions to the MBE (or ≥4-body for 3b:Mb) by means of a computation on the entire cluster. Analytic gradients were used for all geometry optimizations along with analytic Hessians for all harmonic vibrational frequency computations. MP2 computations were carried out using Gaussian16 while all CCSD(T) computations were performed with CFOUR [149,150].

The relative electronic energies (∆E) of the various complexes were calculated by comparing the total energies at each level of theory. The supramolecular approach was used to determine the MP2, 2b:Mb, and 3b:Mb binding energies ($E_{bind}$) and interaction energies ($E_{int}$) of the Ar atom to various water cluster isomers as shown in Equation (1).

(1) $$E_{bind/int}=E\left[\mathrm{Ar}{\left(H_{2}O\right)}_{n}\right]-E\left[{\left(H_{2}O\right)}_{n}\right)]-E\left[\mathrm{Ar}\right]$$

$E_{bind}$ is obtained when $E[{\left(H_{2}O\right)}_{n}]$ is evaluated using the fully optimized geometry of the isolated water cluster, whereas use of the geometry adopted in the full complex yields $E_{int}$. The effects of the harmonic zero-point vibrational energy (ZPVE) were also assessed for all minima, and the ZPVE-inclusive relative and binding energies are denoted ∆E${}^{0}$ and $E_{bind}^{0}$  respectively.

By comparing the total energy of a complex to the sum of fragment energies computed with finite basis sets, Equation (1) introduces an inconsistency commonly referred to as basis set superposition error (BSSE) [151,152]. To assess the potential effects of this inconsistency, the Boys–Bernardi counterpoise (CP) procedure [153,154] was employed to compute for the MP2/haTZ-optimized Ar(H${}_{2}$O)${}_{n=3–5}$ structures. This analysis utilized the protocol outlined elsewhere [155], which corresponds to the default CP scheme in Gaussian16, where the energies of the last two terms in Equation (1) are evaluated in the basis set of the entire cluster.

An additional analysis of the total interaction energies based on symmetry-adapted perturbation theory (SAPT) [156,157,158] was carried out on the 3b:Mb-optimized Ar(H${}_{2}$O)${}_{n=3–5}$ structures. We used the higher-order SAPT2+3(CCD) method that includes a treatment of dispersion based on coupled-cluster doubles and has been shown to provide improvements for challenging cases such as the PCCP dimer [159,160]. The SAPT2+3(CCD) computations were carried out with the haTZ basis set using the efficient implementation in the PSI4 [161,162] quantum chemistry software package that employs natural orbital truncation [163]. Rather than just calculating the total interaction energies as described above, SAPT provides additional insight into how Ar interacts with the small water clusters by identifying the individual contributions from exchange repulsion, electrostatics, induction, and dispersion.

## 3. Results and Discussion

**Figure 1:**
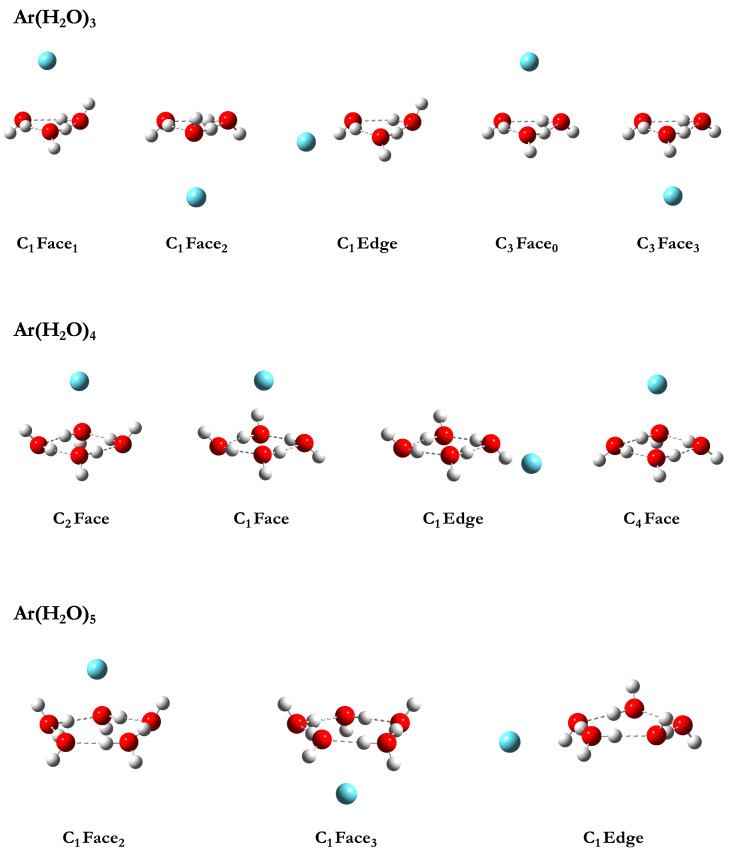
Minima identified for the Ar(H${}_{2}$O)${}_{n=3–5}$ complexes (H white; O red; Ar cyan).

Twelve low-lying stationary points were identified for the Ar(H${}_{2}$O)${}_{n=3–5}$ systems via full geometry optimizations using the haTZ basis set in conjunction with the MP2, 2b:Mb, and 3b:Mb methods, and these structures are shown in Figure 1. Both the faces and edges of small, cyclic water clusters were identified as favorable binding sites for a single Ar atom. These H${}_{2}$O stationary points include the well-characterized C${}_{1}$ and C${}_{3}$ trimers, S${}_{4}$, C${}_{i}$ and C${}_{4}$ tetramers, and C${}_{1}$ pentamer. The distance between the Ar atom and the corresponding face or edge binding site ranges from approximately 3.4 to 3.7 Å across the various Ar(H${}_{2}$O)${}_{n=3–5}$ binding motifs. Harmonic vibrational frequency computations confirm that these Ar(H${}_{2}$O)${}_{n=3–5}$ stationary points are minima at all levels of theory presented in this work. Cartesian coordinates and harmonic vibrational frequencies for all identified structures are reported in the Supporting Information (Table S1–Table S36).

The naming scheme shown in Figure 1 beneath each structure includes the point group symmetry of the Ar(H${}_{2}$O)${}_{n}$ cluster, the binding site of the Ar atom on the (H${}_{2}$O)${}_{n}$ cluster (face or edge) and the number of free hydrogens pointing towards the Ar atom when additional distinction is needed. For example, the first two structures listed at the top left of Figure 1 for Ar(H${}_{2}$O)${}_{3}$ (C${}_{1}$ Face${}_{1}$ and C${}_{1}$ Face${}_{2}$, respectively) both have C${}_{1}$ symmetry and Ar bound to the face of the water trimer. A subscript of 1 is added to the first structure name to indicate one free hydrogen pointing towards the Ar atom, while a subscript of 2 is added to the second structure name to indicate two free hydrogens pointing toward the Ar atom. This distinction is only necessary for some of the Ar(H${}_{2}$O)${}_{3}$ and Ar(H${}_{2}$O)${}_{5}$ clusters.

### 3.1. Structures, Harmonic Vibrational Frequencies, and Relative Energies

Table 1 reports the relative electronic and ZPVE-inclusive energies (∆E and ∆E${}^{0}$, respectively) obtained with the haTZ basis set and the MP2, 2b:Mb, and 3b:Mb methods for all Ar(H${}_{2}$O)${}_{n=3–5}$ minima depicted in Figure 1. The reference values of 0.00 kJ mol${}^{-1}$ correspond to the lowest-energy structure for each Ar(H${}_{2}$O)${}_{n}$ (*n* = 3, 4 and 5) cluster, each of which is depicted in the leftmost image of each row in Figure 1). The first five rows of Table 1 also include ∆E and ∆E${}^{0}$ values for the bare C${}_{1}$ and C${}_{3}$ water trimers and S${}_{4}$, C${}_{i}$ and C${}_{4}$ water tetramers for reference. The haTZ relative electronic and ZPVE-inclusive energies reported in Table 1 are remarkably consistent between all three methods utilized in this work. The 2b:Mb values differ only slightly from the 3b:Mb results (average absolute deviation of 0.06 kJ mol${}^{-1}$ and never by more than ±0.32 kJ mol${}^{-1}$). The deviations from the 3b:Mb ∆E and ∆E${}^{0}$ values tend to be slightly larger for the MP2 method, but they always fall within ±0.43 kJ mol${}^{-1}$.

Note that ∆E${}^{0}$ values are not provided for the C${}_{4}$ bare (H${}_{2}$O)${}_{4}$ tetramer because it is a transition state (denoted by the superscript † symbol) with 1 imaginary vibrational frequency at all three levels of theory. The MP2, 2b:Mb, and 3b:Mb harmonic vibrational frequency computations with the haTZ basis set confirm that all of the other (H${}_{2}$O)${}_{n=3–5}$ and Ar(H${}_{2}$O)${}_{n=3–5}$ stationary points listed in Table 1 are minima. Shifts in the harmonic OH stretching frequencies induced by the binding of an Ar atom to a water trimer, tetramer, or pentamer (Ar(H${}_{2}$O)${}_{n=3–5}$) relative to the OH stretching frequencies of the isolated water cluster ((H${}_{2}$O)${}_{n=3–5}$) were also analyzed. The formation of the Ar(H${}_{2}$O)${}_{n=3–5}$ complexes induces small shifts to lower energy (typically just 1 or 2 cm${}^{-1}$) for every intramolecular vibrational mode relative to the isolated water clusters. However, the shifts grow as large as −5 to −7 cm${}^{-1}$ for some of the hydrogen-bonded OH stretching frequencies when a single Ar atom binds to the face of these cyclic (H${}_{2}$O)${}_{n=3–5}$ clusters. For comparison, the analogous experimental shifts induced by cryogenic Ar matrices and Ar nanocoatings range from approximately −15 to −35 cm${}^{-1}$. (see Table II from Ref. [96]). The shifts predicted with the haTZ basis set are quite consistent across the MP2, 2b:Mb, and 3b:Mb CCSD(T):MP2 methods, and the harmonic vibrational frequencies are reported in the Supporting Information for all Ar(H${}_{2}$O)${}_{n=3–5}$ complexes identified in this work.

#### 3.1.1. Ar(H${}_{2}$O)${}_{3}$

Five structures were identified as minima for the Ar(H${}_{2}$O)${}_{3}$ system in which Ar binds to either a face or an edge of the C${}_{1}$ and C${}_{3}$ water trimer isomers. All unique faces and edges were tested as potential binding sites for a single Ar atom, but the subsequent geometry optimizations always collapsed to one of the five structures reported here. The five binding motifs are shown in the first row of Figure 1; to the best of our knowledge, the rightmost C${}_{3}$ Face${}_{3}$ configuration is the only one that has been previously reported in the literature [95].

Ar binds to both unique faces of the C${}_{1}$ water trimer, as well as the edge in which both free hydrogens are oriented in the same direction. Ar also binds to both unique faces of the C${}_{3}$ water trimer, but does not bind to an edge. All five identified minima are separated by only a few kJ mol${}^{-1}$ at all three levels of theory, as can be seen from the ∆E and ∆E${}^{0}$ data near the middle of Table 1. The structure with the lowest energy has the single Ar on the face of the C${}_{1}$ trimer with only one free hydrogen oriented towards the Ar atom (leftmost image in Figure 1). However, the structure with Ar bound to the other face (C${}_{1}$ Face${}_{2}$) is only higher in energy by a few tenths of a kJ mol${}^{-1}$. The energy increases more significantly when Ar binds to an edge of the cyclic water trimer in the C${}_{1}$ Edge structure, where ∆E grows to more than 1.2 kJ mol${}^{-1}$. The C${}_{3}$ Face${}_{0}$ and C${}_{3}$ Face${}_{3}$ structures also have the largest relative energies, but this difference does not necessarily indicate weak binding (which will be discussed in greater detail in Section 3.2). It is almost entirely due to the underlying energy difference between the C${}_{1}$ and C${}_{3}$ isomers of the water trimer as shown in the first two rows of Table 1.

#### 3.1.2. Ar(H${}_{2}$O)${}_{4}$

The second row of Figure 1 depicts the four minima identified for the Ar(H${}_{2}$O)${}_{4}$ system in which Ar binds to the S${}_{4}$, C${}_{i}$ and C${}_{4}$ cyclic structures of (H${}_{2}$O)${}_{4}$. To the best of our knowledge, none of these complexes have been previously reported. The structure with the lowest energy has the Ar atom on the face of the S${}_{4}$ global minimum of (H${}_{2}$O)${}_{4}$, which results in an Ar(H${}_{2}$O)${}_{4}$ complex with C${}_{2}$ symmetry (leftmost image in the second row of Figure 1). No minima were identified with Ar binding to the edge of the S${}_{4}$ water tetramer at the levels of theory used in this work. However, when Ar is in the presence of the C${}_{i}$ (H${}_{2}$O)${}_{4}$ structure, minima were identified with Ar bound not only to the face but also to the edge with both free hydrogens oriented in the same direction (analogous to the situation for the Ar(H${}_{2}$O)${}_{3}$ system). The resulting C${}_{1}$ Face and C${}_{1}$ Edge Ar(H${}_{2}$O)${}_{4}$ complexes have electronic energies higher than the C${}_{2}$ Face minimum by at least 3.5 and 5.6 kJ mol${}^{-1}$, respectively.

The highest-energy minimum identified (rightmost image in the second row of Figure 1) involves Ar binding to the face of the C${}_{4}$ water tetramer structure with all free hydrogens on the opposite side of the ring, which gives an Ar(H${}_{2}$O)${}_{4}$ complex that maintains C${}_{4}$ symmetry. Interestingly, the C${}_{4}$ structure of the isolated (H${}_{2}$O)${}_{4}$ cluster is a transition state even though the corresponding complex with an Ar atom is a minimum at each level of theory reported here. Furthermore, scans of the Ar atom moving along the C${}_{4}$ axis on the side of the ring with the four free H atoms yielded repulsive potential energy curves. As can be seen from the C${}_{4}$ Face Ar(H${}_{2}$O)${}_{4}$ row of data in in Table 1, the 2b:Mb and 3b:Mb ∆E results grow beyond 8.7 kJ mol${}^{-1}$. As noted for the water trimer systems, however, large ∆E and ∆E${}^{0}$ values do not necessarily indicate weak interactions between the Ar atom and the water cluster. (see Section 3.2). The large relative energies for C${}_{4}$ Face Ar(H${}_{2}$O)${}_{4}$ primarily reflect that the C${}_{4}$ transition state of (H${}_{2}$O)${}_{4}$ has an electronic energy approximately 9 kJ mol${}^{-1}$ higher than the S${}_{4}$ global minimum structure of the water tetramer (fifth row of data in Table 1).

#### 3.1.3. Ar(H${}_{2}$O)${}_{5}$

The last row of Figure 1 shows the three Ar(H${}_{2}$O)${}_{5}$ minima identified with the MP2, 2b:Mb, and 3b:Mb methods in conjunction with the haTZ basis set. The two unique faces of the cyclic water pentamer provide similar binding sites for the Ar atom, but the electronic energy is slightly lower when it binds to the side with two free hydrogens (leftmost image in bottom row of Figure 1) rather than three (middle image in bottom row of Figure 1). The 3b:Mb ∆E for the latter (Ar(H${}_{2}$O)${}_{5}$ C${}_{1}$ Face${}_{3}$) is only 0.36 kJ mol${}^{-1}$. A minimum with Ar bound to an edge was also identified. As with the water trimer and tetramer clusters, a minimum for this motif was only found along the edge with both free hydrogens pointing to the same side of the (H${}_{2}$O)${}_{n}$ ring. To the best of our knowledge, all three binding motifs are reported here for the first time. The last row of Table 1 shows the C${}_{1}$ Edge Ar(H${}_{2}$O)${}_{5}$ structure is noticeably higher in energy compared to the C${}_{1}$ Face${}_{2}$ minimum, with both ∆E and ∆E${}^{0}$ growing larger than 2.3 kJ mol${}^{-1}$. Binding sites on the cyclic C${}_{5}$ (H${}_{2}$O)${}_{5}$ pentamer, analogous to those for the C${}_{3}$ trimer and C${}_{4}$ tetramer, were also investigated. However, all attempts to identify minima on the corresponding faces and edges collapsed to one of the C${}_{1}$ structures shown in the bottom of row of Figure 1.

### 3.2. Binding and Interaction Energies

#### 3.2.1. Binding Energies

The electronic and ZPVE-inclusive binding energies ($E_{bind}$ and $E_{bind}^{0}$) of the haTZ optimized Ar(H${}_{2}$O)${}_{n=3–5}$ minima are reported in Table 2 for the MP2, 2b:Mb, and 3b:Mb methods. Note that $E_{bind}^{0}$ values are not provided for the C${}_{4}$ Ar(H${}_{2}$O)${}_{4}$ complex as the bare (H${}_{2}$O)${}_{4}$ tetramer fragment is a transition state at the associated levels of theory. All three methods are in remarkably good agreement for both quantities. The MP2 and 2b:Mb values (left and middle columns of Table 2) deviate by less than 0.3 kJ mol${}^{-1}$ from the corresponding 3b:Mb $E_{bind}$ and $E_{bind}^{0}$ data in the last two columns of Table 2. For comparison, the electronic binding energy of the ArH${}_{2}$O dimer computed with the same procedures is approximately −1.2 kJ mol${}^{-1}$.

Overall, the tabulated $E_{bind}$ and $E_{bind}^{0}$ data show that Ar binds more strongly to the cyclic water clusters as the size increases from n=3 (top 3 rows of data in Table 2) to n=5 (bottom 3 rows of data in Table 2). Although the enhancement is quite modest when Ar binds to the edge of the cluster (less than 0.5 kJ mol${}^{-1}$), the binding of Ar to a face of C${}_{1}$ (H${}_{2}$O)${}_{5}$ is approximately 2 kJ mol${}^{-1}$ stronger than to a face of C${}_{1}$ (H${}_{2}$O)${}_{3}$. The 3b:Mb $E_{bind}$ values in Table 2 clearly show that Ar binds more strongly to the faces of the water clusters than the edges, and due to the aforementioned trends, the energetic advantage of binding to a face becomes more pronounced as the cluster size increases (from 1.26 kJ mol${}^{-1}$ (or 41%) for n=3 to 2.07 and 2.71 kJ mol${}^{-1}$ (or 50% and 56%) for n=4 and 5). The C${}_{1}$ Ar(H${}_{2}$O)${}_{5}$ Face${}_{2}$ and Face${}_{3}$ complexes exhibit the strongest binding energies out of all of the Ar(H${}_{2}$O)${}_{n=3–5}$ minima identified in this work with $E_{bind}$ exceeding −5.3 kJ mol${}^{-1}$ and approaching −5.8 kJ mol${}^{-1}$. The electronic binding energies indicate that Ar binds slightly more strongly to faces that have fewer free hydrogens oriented towards the Ar atom (by approximately 0.3 kJ mol${}^{-1}$ for C${}_{1}$ Face${}_{1}$ vs. Face${}_{2}$ Ar(H${}_{2}$O)${}_{3}$, 0.8 kJ mol${}^{-1}$ for C${}_{3}$ Face${}_{0}$ vs. Face${}_{3}$ Ar(H${}_{2}$O)${}_{3}$, and 0.4 kJ mol${}^{-1}$ for C${}_{1}$ Face${}_{2}$ vs. Face${}_{3}$ Ar(H${}_{2}$O)${}_{5}$).

When the CP procedure was employed to evaluate the potential impact of the BSSE on the binding energies for the Ar(H${}_{2}$O)${}_{n=3–5}$ minima identified in this work, the MP2/haTZ binding energies were found to decrease in magnitude by approximately 1.1 kJ mol${}^{-1}$ on average and never by more than 1.5 kJ mol${}^{-1}$ for all 12 configurations. These relatively small differences suggest that the results presented in Table 2 are only slightly larger in magnitude that the corresponding values evaluated at the complete basis set limit, where by definition, the BSSE vanishes. All binding energies obtained with the CP procedure can be found in the Supporting Information (Tables S37–S39).

#### 3.2.2. Interaction Energies

The first three columns of Table 3 report the interaction energies ($E_{int}$ in kJ mol${}^{-1}$) calculated for the haTZ optimized Ar(H${}_{2}$O)${}_{n=3–5}$ minima depicted in Figure 1 using the MP2, 2b:Mb and 3b:Mb methods, respectively. The remaining columns report the individual energy components of and the total interaction energy (in kJ mol${}^{-1}$) obtained from SAPT2+3(CCD) computations with the haTZ basis set for the 3b:Mb/haTZ-optimized Ar(H${}_{2}$O)${}_{n=3–5}$ minima. Utilizing SAPT to compute the total interaction energy directly provides the physical contributions from exchange repulsion, electrostatics, induction and dispersion, which are reported in the last four columns of Table 3, respectively.

The MP2, 2b:Mb, and 3b:Mb $E_{int}$ values reported in the left half of Table 3 are in remarkably good agreement with the corresponding binding energies reported in Table 2, with differences never exceeding 0.14 kJ mol${}^{-1}$ across all of the different structures examined and methods utilized in this study. The consistency between $E_{int}$ and $E_{bind}$ values suggest that the binding of an Ar atom to a cyclic water trimer, tetramer or pentamer does not induce any significant geometric changes to the (H${}_{2}$O)${}_{n}$ cluster itself. This observation is consistent with the small perturbations to the intramolecular vibrational frequencies that occur upon binding as noted in Section 3.1.

While the SAPT2+3(CCD) interaction energy values in Table 3 are somewhat smaller in magnitude than the MP2, 2b:Mb, and 3b:Mb $E_{int}$ values, they are also slightly larger than the corresponding MP2 results obtained with the CP procedure that are tabulated in the Supporting Information (Tables S37–S39) which is to be expected because SAPT does not suffer from the BSSE issues introduced via Equation (1). All computations reveal stronger interactions for complexes in which Ar is bound to a face of the water cluster rather than an edge, and the SAPT analysis provides some insight into the underlying factors. The penultimate column of data in Table 3, for example, shows that induction consistently has the smallest contribution to $E_{int}$. Additionally, the attractive induction component is only slightly smaller in magnitude for the C${}_{1}$ Edge structures than the analogous C${}_{1}$ Face minima (by ca. 0.1 to 0.3 kJ mol${}^{-1}$). The electrostatic contributions, which include short-range terms from the overlap of the electron cloud of Ar with that of the water cluster, are larger than those from induction and also favor the face-binding motifs over the edge-binding ones by approximately 0.6 to 1.3 kJ mol${}^{-1}$. In all cases, dispersion (last column of Table 3) is the dominant attractive contribution to $E_{int}$ for these systems in which Ar binds to the edge or face of a cyclic water trimer, tetramer or pentamer. The dispersion components from the SAPT2+3(CCD) computations also exhibit the largest energetic differences between the C${}_{1}$ Edge and corresponding C${}_{1}$ Face structures, being more attractive in the latter by ca. 2 to 5 kJ mol${}^{-1}$. Although all attractive contributions from the SAPT analysis (electrostatics, induction and dispersion) favor the face-binding motifs, the situation is reversed for exchange repulsion, which is smaller for the C${}_{1}$ Edge structures than the corresponding C${}_{1}$ Face motifs by approximately 2 to 4 kJ mol${}^{-1}$. Nevertheless, the contributions from exchange repulsion are not enough to offset the attractive components, and the total SAPT2+3(CCD) $E_{int}$ values are larger in magnitude for the lowest-energy C${}_{1}$ Face minima of the Ar(H${}_{2}$O)${}_{n}$ clusters than the C${}_{1}$ Edge structures by 1.09, 1.80 and 2.41 kJ mol${}^{-1}$ for n=3,4,5, respectively.

## 4. Conclusions

This work systematically identifies the energetically favorable binding sites of a single Ar atom to the well-characterized structures of the cyclic (H${}_{2}$O)${}_{n=3–5}$ trimer, tetramer and pentamer clusters using the haTZ basis set and a variety of methods including MP2 and the highly efficient and accurate 2b:Mb and 3b:Mb methods. Twelve unique Ar(H${}_{2}$O)${}_{n=3–5}$ stationary points have been identified in which Ar binds to either a face or an edge on the water cluster via full geometry optimizations and have been confirmed as minima by harmonic vibrational frequency computations at all three levels of theory. Five, four and three unique stationary points have been identified in which Ar binds to the C${}_{1}$ and C${}_{3}$ water trimers, S${}_{4}$, C${}_{i}$ and C${}_{4}$ water tetramers and C${}_{1}$ water pentamer, respectively. To the best of our knowledge, all of these structures are characterized here for the first time with the exception of the C${}_{3}$ Face${}_{3}$ complex. [95]

Although multiple minima were identified with the Ar bound to a face of each water cluster (9 total), only a single minimum was identified for each value of *n* with Ar bound to an edge (3 total). In every case, a face provided a more energetically favorable binding site for the Ar atom than an edge of the same (H${}_{2}$O)${}_{n}$ cluster. Relative electronic energies (with and without ZPVE correction) ranged from approximately 1 to 3 kJ mol${}^{-1}$ higher in energy for the latter complexes. The binding energies ($E_{bind}$ and $E_{bind}^{0}$) also show that Ar consistently binds more strongly to the faces of the water clusters than the edges (by ca. 1 to 3 kJ mol${}^{-1}$).

The MP2, 2b:Mb, and 3b:Mb electronic interaction energies ($E_{int}$) computed with the haTZ basis set are nearly identical to the corresponding $E_{bind}$ values, suggesting that the binding of an Ar atom has no significant effect on the geometries of the bare (H${}_{2}$O)${}_{n=3–5}$ clusters. The small differences between $E_{bind}$ and $E_{int}$ are also consistent with the very small changes to (nearly) all intramolecular harmonic vibrational frequencies of the water clusters after Ar binds to an edge or face. However, the frequency shifts can be on the order of −5 cm${}^{-1}$ for some of the hydrogen-bonded OH stretching frequencies when a single Ar atom binds to a face of these small cyclic water clusters.

The haTZ total interaction energies computed with SAPT2+3(CCD) qualitatively support these findings, resulting in stronger interaction energies for complexes with Ar bound to a face rather than an edge, with the differences between the SAPT2+3(CCD) values exceeding 2 kJ mol${}^{-1}$ between the two potential binding sites. Notably, using SAPT to compute the total interaction energy provides further insight into the nature of these favorable interactions between Ar and small cyclic water clusters by providing a breakdown of the total interaction energy into physically meaningful components (electrostatics, exchange repulsion, induction and dispersion).

SAPT2+3(CCD) computations with the haTZ basis set indicate that dispersion overwhelmingly provides the dominant attractive component to $E_{int}$ in all cases. It also exhibited the greatest difference between the edge- and face-binding motifs, favoring the latter by just over 2 kJ mol${}^{-1}$ for the trimer and growing to more than 5 kJ mol${}^{-1}$ for the pentamer.

The results presented here for 2-dimensional hydrogen-bonded networks provide some guidelines for the expected binding patterns that Ar will exhibit in larger 3-dimensional water clusters. In the case of (H${}_{2}$O)${}_{6}$, for example, an Ar atom is expected to preferentially bind to the faces rather than the edges edges of the low-lying minima (prism, cage, etc.). Additionally, the interaction strength should increase with the size of the face (from triangular to rectangular and pentagonal), but the overall perturbations to the relative energetics and vibrational frequencies of the hexamer isomers will likely remain quite small and similar in magnitude to those reported here.

## Figures and Tables

**Table 1:** Relative electronic and ZPVE-inclusive energies (∆E and ∆E${}^{0}$, respectively) in kJ mol${}^{-1}$ obtained for the haTZ-optimized (H${}_{2}$O)${}_{n=3,4}$ and Ar(H${}_{2}$O)${}_{n=3–5}$ structures using the MP2, 2b:Mb, and 3b:Mb methods.

		MP2		2b:Mb		3b:Mb	
**Complex**	**Label**	∆E	∆E ${}^{0}$	∆E	∆E ${}^{0}$	∆E	∆E ${}^{0}$
(H${}_{2}$O)${}_{3}$	C${}_{1}$	0.00	0.00	0.00	0.00	0.00	0.00
(H${}_{2}$O)${}_{3}$	C${}_{3}$	3.24	1.72	3.45	1.97	3.43	1.95
(H${}_{2}$O)${}_{4}$	S${}_{4}$	0.00	0.00	0.00	0.00	0.00	0.00
(H${}_{2}$O)${}_{4}$	C${}_{i}$	3.88	2.96	3.92	3.00	3.91	2.98
(H${}_{2}$O)${}_{4}$	C${{}_{4}}^{†}$	9.11	… ${}^{a}$	9.34	… ${}^{a}$	9.31	… ${}^{a}$
Ar(H${}_{2}$O)${}_{3}$	C${}_{1}$ Face${}_{1}$	0.00	0.00	0.00	0.00	0.00	0.00
Ar(H${}_{2}$O)${}_{3}$	C${}_{1}$ Face${}_{2}$	0.36	0.23	0.27	0.17	0.27	0.17
Ar(H${}_{2}$O)${}_{3}$	C${}_{1}$ Edge	1.34	0.90	1.39	0.98	1.26	0.87
Ar(H${}_{2}$O)${}_{3}$	C${}_{3}$ Face${}_{0}$	3.10	1.75	3.40	2.00	3.37	1.98
Ar(H${}_{2}$O)${}_{3}$	C${}_{3}$ Face${}_{3}$	4.17	2.28	4.18	2.41	4.16	2.39
Ar(H${}_{2}$O)${}_{4}$	C${}_{2}$ Face ${}^{b}$	0.00	0.00	0.00	0.00	0.00	0.00
Ar(H${}_{2}$O)${}_{4}$	C${}_{1}$ Face ${}^{c}$	3.50	2.70	3.55	2.73	3.56	2.74
Ar(H${}_{2}$O)${}_{4}$	C${}_{1}$ Edge ${}^{c}$	5.70	4.50	5.88	4.65	5.63	4.43
Ar(H${}_{2}$O)${}_{4}$	C${}_{4}$ Face	8.30	5.50	8.75	5.82	8.73	5.77
Ar(H${}_{2}$O)${}_{5}$	C${}_{1}$ Face${}_{2}$	0.00	0.00	0.00	0.00	0.00	0.00
Ar(H${}_{2}$O)${}_{5}$	C${}_{1}$ Face${}_{3}$	0.49	0.30	0.36	0.22	0.36	0.22
Ar(H${}_{2}$O)${}_{5}$	C${}_{1}$ Edge	2.90	2.44	3.03	2.58	2.71	2.31

${}^{a}$ C${{}_{4}}^{†}$ (H${}_{2}$O)${}_{4}$ is a transition state (harmonic vibrational frequencies in Supporting Information); ${}^{b}$ Ar bound to S${}_{4}$ (H${}_{2}$O)${}_{4}$; ${}^{c}$ Ar bound to C${}_{i}$ (H${}_{2}$O)${}_{4}$.

**Table 2:** Electronic and ZPVE-inclusive binding energies ($E_{bind}$ and $E_{bind}^{0}$, respectively) in kJ mol${}^{-1}$ for the haTZ-optimized Ar(H${}_{2}$O)${}_{n=3–5}$ complexes with the MP2, 2b:Mb, and 3b:Mb methods.

	MP2		2b:Mb		3b:Mb	
**Label**	$E_{\mathrm{bind}}$	$E_{\mathrm{bind}}^{0}$	$E_{\mathrm{bind}}$	$E_{\mathrm{bind}}^{0}$	$E_{\mathrm{bind}}$	$E_{\mathrm{bind}}^{0}$
Binding Process: C${}_{1}$ (H${}_{2}$O)${}_{3}$ + Ar → C${}_{1}$ Ar(H${}_{2}$O)${}_{3}$						
C${}_{1}$ Face${}_{1}$	−3.88	−3.10	−3.99	−3.21	−3.84	−3.09
C${}_{1}$ Face${}_{2}$	−3.52	−2.87	−3.73	−3.04	−3.57	−2.92
C${}_{1}$ Edge	−2.54	−2.20	−2.60	−2.23	−2.58	−2.23
Binding Process: C${}_{3}$ (H${}_{2}$O)${}_{3}$ + Ar → C${}_{3}$ Ar(H${}_{2}$O)${}_{3}$						
C${}_{3}$ Face${}_{0}$	−4.02	−3.07	−4.04	−3.18	−3.90	−3.06
C${}_{3}$ Face${}_{3}$	−2.95	−2.53	−3.26	−2.77	−3.11	−2.65
Binding Process: S${}_{4}$ (H${}_{2}$O)${}_{4}$ + Ar → C${}_{2}$ Ar(H${}_{2}$O)${}_{4}$						
C${}_{2}$ Face	−4.65	−3.92	−4.84	−4.08	−4.62	−3.90
Binding Process: C${}_{i}$ (H${}_{2}$O)${}_{4}$ + Ar → C${}_{1}$ Ar(H${}_{2}$O)${}_{4}$						
C${}_{1}$ Face	−5.03	−4.18	−5.21	−4.34	−4.98	−4.16
C${}_{1}$ Edge	−2.82	−2.39	−2.88	−2.42	−2.91	−2.46
Binding Process: C${{}_{4}}^{†}$ (H${}_{2}$O)${}_{4}$ + Ar → C${}_{4}$ Ar(H${}_{2}$O)${}_{4}$						
C${}_{4}$ Face	−5.46	… ${}^{a}$	−5.43	… ${}^{a}$	−5.20	… ${}^{a}$
Binding Process: C${}_{1}$ (H${}_{2}$O)${}_{5}$ + Ar → C${}_{1}$ Ar(H${}_{2}$O)${}_{5}$						
C${}_{1}$ Face${}_{2}$	−5.85	−4.92	−6.03	−5.09	−5.75	−4.86
C${}_{1}$ Face${}_{3}$	−5.36	−4.61	−5.67	−4.87	−5.39	−4.64
C${}_{1}$ Edge	−2.95	−2.47	−3.01	−2.51	−3.04	−2.55

${}^{a}$ C${{}_{4}}^{†}$ (H${}_{2}$O)${}_{4}$ is a transition state (harmonic vibrational frequencies in Supporting Information).

**Table 3:** Interaction energies ($E_{int}$ in kJ mol${}^{-1}$) calculated for the Ar(H${}_{2}$O)${}_{n=3–5}$ minima using the MP2, 2b:Mb, and 3b:Mb methods with the haTZ basis set as well as the SAPT2+3(CCD) total interaction energies computed with the haTZ basis set for the 3b:Mb/haTZ optimized structures followed by the individual contributions from exchange repulsion, electrostatics, induction, and dispersion (in kJ mol${}^{-1}$).

	$E_{\mathrm{int}}$				SAPT Components			
**Label**	**MP2**	**2b:Mb**	**3b:Mb**	**SAPT**	**Exch**	**Elect**	**Ind**	**Disp**
Binding Process: C${}_{1}$ (H${}_{2}$O)${}_{3}$ + Ar → C${}_{1}$ Ar(H${}_{2}$O)${}_{3}$								
C${}_{1}$ Face${}_{1}$	−3.90	−4.00	−3.85	−3.13	+5.62	−1.73	−0.66	−6.37
C${}_{1}$ Face${}_{2}$	−3.53	−3.74	−3.58	−2.92	+5.42	−1.71	−0.55	−6.08
C${}_{1}$ Edge	−2.54	−2.60	−2.58	−2.04	+3.66	−1.09	−0.40	−4.22
Binding Process: C${}_{3}$ (H${}_{2}$O)${}_{3}$ + Ar → C${}_{3}$ Ar(H${}_{2}$O)${}_{3}$								
C${}_{3}$ Face${}_{0}$	−4.03	−4.04	−3.90	−3.08	+5.41	−1.63	−0.53	−6.33
C${}_{3}$ Face${}_{3}$	−2.95	−3.26	−3.11	−2.43	+4.96	−1.60	−0.20	−5.59
Binding Process: S${}_{4}$ (H${}_{2}$O)${}_{4}$ + Ar → C${}_{2}$ Ar(H${}_{2}$O)${}_{4}$								
C${}_{2}$ Face	−4.71	−4.90	−4.66	−3.71	+6.94	−2.17	−0.39	−8.08
Binding Process: C${}_{i}$ (H${}_{2}$O)${}_{4}$ + Ar → C${}_{1}$ Ar(H${}_{2}$O)${}_{4}$								
C${}_{1}$ Face	−5.05	−5.23	−4.99	−4.08	+7.20	−2.23	−0.77	−8.28
C${}_{1}$ Edge	−2.83	−2.89	−2.91	−2.28	+4.05	−1.23	−0.44	−4.65
Binding Process: C${{}_{4}}^{†}$ (H${}_{2}$O)${}_{4}$ + Ar → C${}_{4}$ Ar(H${}_{2}$O)${}_{4}$								
C${}_{4}$ Face	−5.48	−5.44	−5.20	−4.09	+7.30	−2.16	−0.53	−8.69
Binding Process: C${}_{1}$ (H${}_{2}$O)${}_{5}$ + Ar → C${}_{1}$ Ar(H${}_{2}$O)${}_{5}$								
C${}_{1}$ Face${}_{2}$	−5.97	−6.15	−5.84	−4.79	+8.51	−2.64	−0.57	−10.01
C${}_{1}$ Face${}_{3}$	−5.50	−5.81	−5.50	−4.42	+8.29	−2.63	−0.45	−9.62
C${}_{1}$ Edge	−2.96	−3.01	−3.05	−2.38	+4.28	−1.31	−0.49	−4.86

## Data Availability

Data are contained within the article and Supplementary Materials.

## Supplementary Materials

The supporting information can be downloaded at: https://www.mdpi.com/article/10.3390/ijms242417480/s1.
