# Supplementary material for: Insight into the Binding of Argon to Cyclic Water Clusters from Symmetry-Adapted Perturbation Theory
Source: Int J Mol Sci. 2023 Dec 14;24(24):17480. doi: 10.3390/ijms242417480 (PMC10744083; doi:10.3390/ijms242417480)
Supplement: Supplementary file 1 [file ijms-24-17480-s001.zip › ijms-2771681-supplementary.pdf]

Supporting Information for:
Insight into the Binding of Ar to Cyclic Water Clusters from Symmetry-Adapted Perturbation Theory

Carly A. Rock and Gregory S. Tschumper*

Department of Chemistry and Biochemistry

University of Mississippi, University, Mississippi 38677–1848 USA

*Author to whom all correspondence should be addressed.

email: tschumpr@olemiss.edu

ORCID: 0000-0002-3933-2200

CONTENTS

I. Cartesian Coordinates S3

A. $Ar(H_2O)_3$ S3

B. $Ar(H_2O)_4$ S6

C. $Ar(H_2O)_5$ S8

D. $(H_2O)_3$ S10

E. $(H_2O)_4$ S11

F. $(H_2O)_5$ S13

II. Harmonic Vibrational Frequencies S14

A. $Ar(H_2O)_3$ and $(H_2O)_3$ S14

B. $Ar(H_2O)_4$ and $(H_2O)_4$ S21

C. $(H_2O)_5$ and $Ar(H_2O)_5$ S28

III. Counterpoise-Corrected Binding and Interaction Energies S32

I. CARTESIAN COORDINATES

A. $Ar(H_2O)_3$

TABLE S1. Cartesian coordinates in Angstroms (Å) for the C_1 $Ar(H_2O)_3$ $Face_1$ optimized geometry at the 3b:Mb/haTZ level of theory.

Atom	x	y	z
O	-1.147792	-1.182099	1.086931
O	-1.300617	-0.346940	-1.580026
O	-1.095780	1.553084	0.469401
H	-1.768156	2.147901	0.812403
H	-1.169807	0.742405	1.002086
H	-1.826184	-1.783983	1.404617
H	-1.212164	-1.208626	0.115753
H	-0.561537	-0.362006	-2.194540
H	-1.235339	0.510529	-1.124299
Ar	2.007039	-0.013255	0.009641

TABLE S2. Cartesian coordinates in Angstroms (Å) for the C_1 $Ar(H_2O)_3$ $Face_2$ optimized geometry at the 3b:Mb/haTZ level of theory.

Atom	x	y	z
O	-1.317705	-1.452658	-0.707233
O	-1.033187	0.081277	1.621889
O	-1.369139	1.348717	-0.848689
H	-0.720263	1.821594	-1.376696
H	-1.320277	0.423470	-1.145311
H	-0.641265	-2.110925	-0.887419
H	-1.204482	-1.215990	0.230067
H	-1.730779	0.242315	2.263388
H	-1.136022	0.779751	0.951554
Ar	2.028519	0.013394	-0.031296

TABLE S3. Cartesian coordinates in Angstroms (Å) for the C_1 $Ar(H_2O)_3$ Edge optimized geometry at the 3b:Mb/haTZ level of theory.

Atom	x	y	z
O	-0.664697	-1.342769	-0.021570
O	-3.176655	-0.109295	0.032527
O	-0.857272	1.453590	-0.022295
H	-0.439003	1.979438	0.664609
H	-0.458908	0.568967	0.048793
H	-0.479801	-1.998420	0.656159
H	-1.630955	-1.224945	-0.010697
H	-3.766324	0.003077	-0.718089
H	-2.593609	0.669836	0.019034
Ar	2.608755	-0.000565	-0.031617

TABLE S4. Cartesian coordinates in Angstroms (Å) for the C_3 $Ar(H_2O)_3$ $Face_0$ optimized geometry at the 3b:Mb/haTZ level of theory.

Atom	x	y	z
O	0.000000	1.617547	-1.133889
O	1.400837	-0.808774	-1.133889
O	-1.400837	-0.808774	-1.133889
H	-1.911633	-1.330264	-1.758412
H	-0.491076	-1.147216	-1.193612
H	-0.196226	2.320655	-1.758412
H	-0.747980	0.998892	-1.193612
H	2.107859	-0.990391	-1.758412
H	1.239056	0.148324	-1.193612
Ar	0.000000	0.000000	2.003857

TABLE S5. Cartesian coordinates in Angstroms (Å) for the C_3 $Ar(H_2O)_3$ $Face_3$ optimized geometry at the 3b:Mb/haTZ level of theory.

Atom	x	y	z
O	-0.139075	1.611228	-1.302632
O	1.464902	-0.685172	-1.302632
O	-1.325827	-0.926056	-1.302632
H	0.659272	1.058905	-1.245824
H	0.587403	-1.100398	-1.245824
H	-1.246674	0.041494	-1.245824
H	0.000000	2.330148	-0.680915
H	2.017967	-1.165074	-0.680915
H	-2.017967	-1.165074	-0.680915
Ar	0.000000	0.000000	2.057965

B. $Ar(H_2O)_4$

TABLE S6. Cartesian coordinates in Angstroms (Å) for the C_2 $Ar(H_2O)_4$ Face optimized geometry at the 3b:Mb/haTZ level of theory.

Atom	x	y	z
O	1.933655	0.000335	-0.924749
O	0.000000	1.954107	-0.971266
O	-1.933655	-0.000335	-0.924749
O	0.000000	-1.954107	-0.971266
H	1.347873	0.785221	-0.932697
H	-0.126700	2.545599	-0.224335
H	-1.347873	-0.785221	-0.932697
H	0.779406	-1.361264	-0.964742
H	2.536419	0.127514	-1.662556
H	-0.779406	1.361264	-0.964742
H	-2.536419	-0.127514	-1.662556
H	0.126700	-2.545599	-0.224335
Ar	0.000000	0.000000	2.105828

TABLE S7. Cartesian coordinates in Angstroms (Å) for the C_1 $Ar(H_2O)_4$ Face optimized geometry at the 3b:Mb/haTZ level of theory.

Atom	x	y	z
H	0.497598	-1.526856	-2.066822
O	1.163707	-1.222567	-1.444452
H	0.901298	-1.537847	0.290134
H	1.139352	-0.245623	-1.496512
O	0.755590	-1.513527	1.258382
H	1.406537	-2.116975	1.626664
H	0.745199	0.253618	1.604522
O	0.709725	1.230084	1.550601
H	1.358587	1.544614	2.185966
H	0.983725	1.548522	-0.187212
O	1.130875	1.522935	-1.154680
H	0.472843	2.117387	-1.524956
Ar	-2.088017	-0.009569	-0.117255

TABLE S8. Cartesian coordinates in Angstroms (Å) for the C_1 $Ar(H_2O)_4$ Edge optimized geometry at the 3b:Mb/haTZ level of theory.

Atom	x	y	z
H	-3.254569	-1.862007	0.626784
O	-2.737180	-1.465856	-0.079392
H	-0.955850	-1.483683	0.027173
H	-2.952031	-0.511603	-0.055649
O	0.002586	-1.289760	0.077542
H	0.405490	-1.827571	-0.609439
H	0.040669	0.508802	0.054827
O	-0.171299	1.463604	0.087413
H	0.356924	1.865811	-0.607322
H	-1.951959	1.482026	-0.036858
O	-2.909980	1.289102	-0.095590
H	-3.320717	1.843704	0.572931
Ar	3.231057	0.000433	0.005988

TABLE S9. Cartesian coordinates in Angstroms (Å) for the C_4 $Ar(H_2O)_4$ Face optimized geometry at the 3b:Mb/haTZ level of theory.

Atom	x	y	z
O	0.000000	1.952917	-0.859867
O	-1.952917	0.000000	-0.859867
O	0.000000	-1.952917	-0.859867
O	1.952917	0.000000	-0.859867
H	-0.791921	1.380828	-0.897929
H	-1.380828	-0.791921	-0.897929
H	0.791921	-1.380828	-0.897929
H	1.380828	0.791921	-0.897929
H	-0.167524	2.666849	-1.480200
H	-2.666849	-0.167524	-1.480200
H	0.167524	-2.666849	-1.480200
H	2.666849	0.167524	-1.480200
Ar	0.000000	0.000000	2.057126

C. $Ar(H_2O)_5$

TABLE S10. Cartesian coordinates in Angstroms (Å) for the C_1 $Ar(H_2O)_5$ $Face_2$ optimized geometry at the 3b:Mb/haTZ level of theory.

Atom	x	y	z
O	-1.907512	1.454771	-0.517613
H	-0.998749	1.821999	-0.485258
H	-2.310851	1.721256	0.313230
O	0.662827	2.370304	-0.391286
H	1.309774	1.633816	-0.424579
H	0.969203	3.002894	-1.046666
O	2.381438	0.250500	-0.518994
H	1.871417	-0.560007	-0.728999
H	2.869325	0.036126	0.280983
O	0.866778	-1.957039	-1.075822
H	-0.090894	-1.750414	-1.090938
H	1.037681	-2.431031	-1.894017
O	-1.781081	-1.229983	-1.009089
H	-1.863996	-0.268956	-0.830479
H	-2.405982	-1.406952	-1.717205
Ar	-0.120363	-0.494842	1.984798

TABLE S11. Cartesian coordinates in Angstroms (Å) for the C_1 $Ar(H_2O)_5$ $Face_3$ optimized geometry at the 3b:Mb/haTZ level of theory.

Atom	x	y	z
O	-2.349036	0.242725	-0.627864
H	-1.733711	1.005875	-0.657015
H	-2.877964	0.377892	0.163081
O	-0.542451	2.289443	-0.689271
H	0.379646	1.956193	-0.667352
H	-0.570997	2.893555	-1.436307
O	1.997515	1.280686	-0.644343
H	1.982695	0.304183	-0.723267
H	2.522059	1.463243	0.139852
O	1.763146	-1.439062	-0.930257
H	0.816018	-1.692772	-0.918452
H	2.202690	-2.085107	-0.371287
O	-0.900719	-2.059569	-0.881094
H	-1.453891	-1.257233	-0.769994
H	-1.251718	-2.494037	-1.663481
Ar	0.013196	-0.165866	2.060381

TABLE S12. Cartesian coordinates in Angstroms (Å) for the C_1 $Ar(H_2O)_5$ Edge optimized geometry at the 3b:Mb/haTZ level of theory.

Atom	x	y	z
O	3.674936	0.122876	0.214087
H	3.188207	-0.718239	0.083155
H	4.169692	0.003536	1.029090
O	2.210822	-2.155836	-0.135899
H	1.253310	-1.950203	-0.089504
H	2.321072	-2.652153	-0.951405
O	-0.433325	-1.477191	-0.013793
H	-0.547478	-0.504107	-0.024094
H	-0.946829	-1.785359	0.737587
O	-0.576731	1.260903	-0.124201
H	0.324411	1.647729	-0.113105
H	-1.090429	1.803248	0.480034
O	1.973945	2.241121	-0.070567
H	2.614921	1.509852	0.055093
H	2.286200	2.713671	-0.847029
Ar	-3.798348	-0.000165	0.037953

D. $(H_2O)_3$

TABLE S13. Cartesian coordinates in Angstroms (Å) for the C_1 $(H_2O)_3$ optimized geometry at the 3b:Mb/haTZ level of theory.

Atom	x	y	z
O	-1.315032	-0.938073	-0.093648
O	-0.159046	1.603584	0.110193
O	1.475422	-0.658902	-0.084171
H	2.013264	-1.091694	0.584114
H	0.605037	-1.090603	-0.037459
H	-1.971063	-1.183030	0.564156
H	-1.224952	0.028464	-0.022374
H	-0.069914	2.238119	-0.606162
H	0.636869	1.045877	0.058733

TABLE S14. Cartesian coordinates in Angstroms (Å) for the C_3 $(H_2O)_3$ optimized geometry at the 3b:Mb/haTZ level of theory.

Atom	x	y	z
O	-0.969499	1.294050	-0.067204
O	1.605429	0.192586	-0.067204
O	-0.635930	-1.486635	-0.067204
H	0.000000	1.248380	-0.008875
H	1.081128	-0.624190	-0.008875
H	-1.081128	-0.624190	-0.008875
H	-1.233022	1.984303	0.546509
H	2.334968	0.075676	0.546509
H	-1.101946	-2.059979	0.546509

E. $(H_2O)_4$

TABLE S15. Cartesian coordinates in Angstroms (Å) for the S_4 $(H_2O)_4$ optimized geometry at the 3b:Mb/haTZ level of theory.

Atom	x	y	z
O	-1.374549	1.374549	0.010221
O	1.374549	-1.374549	0.010221
O	1.374549	1.374549	-0.010221
O	-1.374549	-1.374549	-0.010221
H	-1.512095	0.405274	-0.006717
H	1.512095	-0.405274	-0.006717
H	0.405274	1.512095	0.006717
H	-0.405274	-1.512095	0.006717
H	-1.877845	1.688729	0.766212
H	1.877845	-1.688729	0.766212
H	1.688729	1.877845	-0.766212
H	-1.688729	-1.877845	-0.766212

TABLE S16. Cartesian coordinates in Angstroms (Å) for the C_i $(H_2O)_4$ optimized geometry at the 3b:Mb/haTZ level of theory.

Atom	x	y	z
H	-0.871619	2.417750	-0.705132
O	-0.589837	1.857200	0.022671
H	1.051947	1.156395	-0.004677
H	-1.168650	1.069352	-0.016723
O	1.854716	0.596137	-0.014370
H	2.418249	0.950344	0.678565
H	1.168650	-1.069352	0.016723
O	0.589837	-1.857200	-0.022671
H	0.871619	-2.417750	0.705132
H	-1.051947	-1.156395	0.004677
O	-1.854716	-0.596137	0.014370
H	-2.418249	-0.950344	-0.678565

TABLE S17. Cartesian coordinates in Angstroms (Å) for the C_4 $(H_2O)_4$ optimized geometry at the 3b:Mb/haTZ level of theory.

Atom	x	y	z
O	1.380356	1.380356	-0.064627
O	1.380356	-1.380356	-0.064627
O	-1.380356	-1.380356	-0.064627
O	-1.380356	1.380356	-0.064627
H	1.537456	0.416370	-0.026017
H	0.416370	-1.537456	-0.026017
H	-1.537456	-0.416370	-0.026017
H	-0.416370	1.537456	-0.026017
H	2.013643	1.770088	0.543037
H	1.770088	-2.013643	0.543037
H	-2.013643	-1.770088	0.543037
H	-1.770088	2.013643	0.543037

F. $(H_2O)_5$

TABLE S18. Cartesian coordinates in Angstroms (Å) for the C_1 $(H_2O)_5$ optimized geometry at the 3b:Mb/haTZ level of theory.

Atom	x	y	z
O	2.316075	-0.088065	0.168376
H	1.751364	-0.880135	0.045126
H	2.818384	-0.258758	0.969492
O	0.640153	-2.219831	-0.155432
H	-0.292898	-1.929186	-0.077221
H	0.679371	-2.718382	-0.976043
O	-1.928009	-1.309801	0.061596
H	-1.957328	-0.330774	0.045246
H	-2.428500	-1.566514	0.840508
O	-1.824140	1.429705	-0.070601
H	-0.891683	1.732920	-0.075391
H	-2.281301	2.032342	0.521529
O	0.805960	2.176345	-0.065013
H	1.381208	1.389759	0.043242
H	1.141077	2.621903	-0.847889

II. HARMONIC VIBRATIONAL FREQUENCIES

A. $Ar(H_2O)_3$ and $(H_2O)_3$

TABLE S19. haTZ harmonic vibrational frequencies (ω in cm^{-1}) for the optimized C_1 $Face_1$ $Ar(H_2O)_3$ complex using the MP2, 2b:Mb and 3b:Mb methods.

Mode	MP2	2b:Mb	3b:Mb
1	34	35	34
2	38	39	37
3	45	46	45
4	180	178	178
5	186	185	184
6	192	191	191
7	206	204	204
8	219	220	219
9	244	244	244
10	349	342	342
11	362	356	356
12	448	440	441
13	576	566	565
14	668	655	655
15	866	856	856
16	1641	1660	1660
17	1645	1663	1663
18	1666	1682	1682
19	3577	3607	3606
20	3641	3663	3663
21	3651	3672	3671
22	3902	3883	3884
23	3905	3887	3888
24	3906	3887	3888

TABLE S20. haTZ harmonic vibrational frequencies (ω in cm^{-1}) for the optimized C_1 $Face_2$ $Ar(H_2O)_3$ complex using the MP2, 2b:Mb and 3b:Mb methods.

Mode	MP2	2b:Mb	3b:Mb
1	29	31	29
2	36	37	36
3	45	46	45
4	174	175	175
5	184	184	183
6	189	189	188
7	203	202	202
8	220	220	219
9	244	242	243
10	348	342	342
11	361	356	356
12	447	440	440
13	575	565	565
14	669	655	656
15	864	855	855
16	1642	1660	1660
17	1644	1663	1662
18	1667	1682	1682
19	3578	3607	3607
20	3641	3664	3663
21	3651	3671	3671
22	3901	3883	3884
23	3907	3888	3888
24	3907	3888	3889

TABLE S21. haTZ harmonic vibrational frequencies (ω in cm^{-1}) for the optimized C_1 Edge $Ar(H_2O)_3$ complex using the MP2, 2b:Mb and 3b:Mb methods.

Mode	MP2	2b:Mb	3b:Mb
1	5	5	5
2	25	26	25
3	39	39	39
4	172	172	172
5	183	183	182
6	189	188	188
7	202	200	200
8	219	220	219
9	241	241	241
10	346	340	340
11	358	352	352
12	447	440	440
13	573	564	564
14	668	655	655
15	860	852	852
16	1641	1660	1660
17	1643	1662	1662
18	1667	1683	1683
19	3581	3610	3609
20	3643	3666	3665
21	3653	3674	3672
22	3905	3885	3886
23	3906	3888	3888
24	3908	3889	3890

TABLE S22. haTZ harmonic vibrational frequencies (ω in cm^{-1}) for the optimized C_3 $Face_0$ $Ar(H_2O)_3$ complex using the MP2, 2b:Mb and 3b:Mb methods.

Mode	MP2	2b:Mb	3b:Mb
1	37	37	36
2	37	37	36
3	45	45	45
4	145	143	143
5	145	143	143
6	186	185	184
7	186	185	184
8	199	197	197
9	218	218	218
10	362	355	355
11	362	355	355
12	515	504	505
13	532	522	522
14	532	522	522
15	808	799	799
16	1647	1665	1665
17	1647	1665	1665
18	1672	1688	1688
19	3601	3628	3627
20	3659	3679	3679
21	3659	3679	3679
22	3910	3892	3893
23	3901	3892	3893
24	3901	3892	3893

TABLE S23. haTZ harmonic vibrational frequencies (ω in cm^{-1}) for the optimized C_3 $Face_3$ $Ar(H_2O)_3$ complex using the MP2, 2b:Mb and 3b:Mb methods.

Mode	MP2	2b:Mb	3b:Mb
1	29	32	30
2	29	32	30
3	39	42	41
4	121	127	126
5	121	127	126
6	185	184	184
7	185	184	184
8	187	190	189
9	217	217	217
10	359	352	352
11	359	352	352
12	516	505	506
13	529	522	521
14	529	522	521
15	798	792	793
16	1646	1664	1664
17	1646	1664	1664
18	1673	1689	1689
19	3604	3629	3628
20	3661	3681	3680
21	3661	3681	3680
22	3912	3893	3894
23	3912	3893	3894
24	3913	3893	3894

TABLE S24. haTZ harmonic vibrational frequencies (ω in cm^{-1}) for the optimized C_1 $(H_2O)_3$ complex using the MP2, 2b:Mb and 3b:Mb methods.

Mode	MP2	2b:Mb	3b:Mb
1	174	174	174
2	182	182	182
3	189	188	188
4	202	200	200
5	219	219	219
6	241	241	241
7	346	340	340
8	359	353	353
9	447	439	439
10	574	564	564
11	669	656	656
12	860	852	852
13	1642	1660	1660
14	1644	1663	1662
15	1668	1683	1683
16	3582	3610	3610
17	3644	3667	3666
18	3654	3674	3674
19	3905	3885	3886
20	3909	3890	3890
21	3909	3890	3891

TABLE S25. haTZ harmonic vibrational frequencies (ω in cm^{-1}) for the optimized C_3 $(H_2O)_3$ complex using the MP2, 2b:Mb and 3b:Mb methods.

Mode	MP2	2b:Mb	3b:Mb
1	127	131	131
2	127	131	131
3	185	184	184
4	185	184	184
5	191	192	192
6	217	217	216
7	359	352	352
8	359	352	352
9	517	506	507
10	529	521	521
11	529	521	521
12	799	794	794
13	1647	1665	1664
14	1647	1665	1664
15	1673	1689	1689
16	3606	3631	3631
17	3663	3682	3682
18	3663	3682	3682
19	3913	3894	3895
20	3913	3894	3895
21	3914	3895	3895

B. $Ar(H_2O)_4$ and $(H_2O)_4$

TABLE S26. haTZ harmonic vibrational frequencies (ω in cm^{-1}) for the optimized C_2 Face $Ar(H_2O)_4$ complex using the MP2, 2b:Mb and 3b:Mb methods.

Mode	MP2	2b:Mb	3b:Mb
1	27	28	26
2	40	41	40
3	43	44	43
4	57	57	56
5	84	81	81
6	209	208	208
7	220	215	215
8	238	235	235
9	242	239	238
10	256	254	253
11	260	256	256
12	260	256	256
13	292	289	289
14	411	401	402
15	434	424	424
16	452	442	442
17	455	445	446
18	757	738	740
19	820	801	801
20	824	805	805
21	983	964	965
22	1646	1666	1665
23	1660	1677	1677
24	1662	1679	1678
25	1690	1704	1704
26	3395	3455	3453
27	3489	3538	3536
28	3493	3541	3539
29	3531	3575	3573
30	3896	3878	3880
31	3897	3879	3880
32	3898	3879	3881
33	3899	3879	3881

TABLE S27. haTZ harmonic vibrational frequencies (ω in cm^{-1}) for the optimized C_1 Face $Ar(H_2O)_4$ complex using the MP2, 2b:Mb and 3b:Mb methods.

Mode	MP2	2b:Mb	3b:Mb
1	33	34	33
2	36	37	36
3	43	42	41
4	47	48	46
5	79	77	77
6	202	197	197
7	206	205	205
8	215	212	211
9	241	238	237
10	245	243	242
11	254	251	250
12	255	254	253
13	271	269	268
14	396	384	385
15	407	398	399
16	494	482	482
17	539	528	529
18	665	648	648
19	750	733	733
20	828	809	810
21	945	928	930
22	1658	1675	1675
23	1659	1677	1677
24	1680	1696	1696
25	1689	1703	1703
26	3410	3467	3466
27	3494	3542	3540
28	3510	3556	3553
29	3540	3582	3581
30	3898	3880	3881
31	3899	3880	3882
32	3900	3881	3882
33	3901	3882	3883

TABLE S28. haTZ harmonic vibrational frequencies (ω in cm^{-1}) for the optimized C_1 Edge $Ar(H_2O)_4$ complex using the MP2, 2b:Mb and 3b:Mb methods.

Mode	MP2	2b:Mb	3b:Mb
1	9	9	9
2	25	25	25
3	32	30	31
4	38	39	39
5	75	74	74
6	195	191	192
7	205	205	204
8	212	209	209
9	240	238	237
10	243	242	241
11	251	249	249
12	255	251	251
13	268	267	266
14	394	382	383
15	405	396	396
16	495	482	483
17	539	528	529
18	665	648	649
19	747	731	732
20	827	809	810
21	942	926	927
22	1657	1675	1675
23	1659	1677	1677
24	1680	1696	1695
25	1690	1704	1704
26	3413	3471	3468
27	3497	3545	3542
28	3512	3558	3555
29	3542	3584	3582
30	3900	3881	3882
31	3901	3882	3883
32	3902	3883	3884
33	3903	3884	3885

TABLE S29. haTZ harmonic vibrational frequencies (ω in cm^{-1}) for the optimized C_4 Face $Ar(H_2O)_4$ complex using the MP2, 2b:Mb and 3b:Mb methods.

Mode	MP2	2b:Mb	3b:Mb
1	29	25	22
2	40	40	38
3	40	40	38
4	47	47	46
5	65	63	63
6	160	156	155
7	160	156	155
8	182	179	179
9	201	200	200
10	213	209	209
11	239	236	235
12	239	236	235
13	247	244	243
14	368	357	357
15	485	473	474
16	485	473	474
17	580	566	568
18	596	579	580
19	710	694	694
20	710	694	694
21	866	852	853
22	1672	1688	1688
23	1672	1688	1688
24	1673	1689	1689
25	1690	1705	1705
26	3447	3501	3499
27	3528	3572	3570
28	3528	3572	3570
29	3561	3600	3598
30	3905	3887	3888
31	3906	3888	3889
32	3906	3888	3889
33	3907	3888	3890

TABLE S30. haTZ harmonic vibrational frequencies (ω in cm^{-1}) for the optimized S_4 $(H_2O)_4$ complex using the MP2, 2b:Mb and 3b:Mb methods.

Mode	MP2	2b:Mb	3b:Mb
1	50	48	49
2	80	78	78
3	208	207	207
4	214	209	210
5	239	236	236
6	239	236	236
7	255	253	252
8	255	253	252
9	260	255	255
10	289	286	286
11	408	399	399
12	436	426	426
13	451	442	442
14	451	442	442
15	754	735	736
16	820	801	802
17	820	801	802
18	984	964	966
19	1647	1666	1666
20	1661	1678	1678
21	1661	1678	1678
22	1689	1704	1704
23	3401	3460	3458
24	3496	3544	3541
25	3496	3544	3541
26	3535	3578	3576
27	3900	3881	3882
28	3900	3882	3883
29	3900	3882	3883
30	3901	3882	3883

TABLE S31. haTZ harmonic vibrational frequencies (ω in cm^{-1}) for the optimized C_i $(H_2O)_4$ complex using the MP2, 2b:Mb and 3b:Mb methods.

Mode	MP2	2b:Mb	3b:Mb
1	31	30	30
2	74	73	73
3	196	192	192
4	205	204	204
5	212	208	208
6	239	237	237
7	243	241	241
8	251	249	249
9	254	250	250
10	267	266	266
11	394	382	383
12	404	395	396
13	493	481	481
14	538	527	528
15	664	647	648
16	747	731	731
17	826	807	808
18	941	925	926
19	1658	1675	1675
20	1660	1677	1677
21	1680	1696	1696
22	1690	1704	1704
23	3415	3472	3470
24	3498	3546	3544
25	3514	3560	3557
26	3544	3585	3583
27	3901	3882	3884
28	3902	3883	3884
29	3903	3884	3885
30	3903	3884	3885

TABLE S32. haTZ harmonic vibrational frequencies (ω in cm^{-1}) for the optimized C_4 $(H_2O)_4$ complex using the MP2, 2b:Mb and 3b:Mb methods.

Mode	MP2	2b:Mb	3b:Mb
1	$44i$	$42i$	$42i$
2	60	59	59
3	141	142	141
4	141	142	141
5	166	167	167
6	198	197	198
7	205	204	204
8	239	236	235
9	239	236	235
10	246	243	243
11	364	354	354
12	483	471	472
13	483	471	472
14	581	567	569
15	594	578	579
16	705	691	691
17	705	691	691
18	857	845	847
19	1672	1688	1688
20	1672	1688	1688
21	1672	1689	1689
22	1691	1706	1706
23	3454	3505	3503
24	3534	3576	3574
25	3534	3576	3574
26	3566	3604	3602
27	3909	3890	3891
28	3910	3891	3892
29	3910	3891	3892
30	3911	3891	3892

C. $(H_2O)_5$ and $Ar(H_2O)_5$

TABLE S33. haTZ harmonic vibrational frequencies (ω in cm^{-1}) for the optimized C_1 $(H_2O)_5$ complex using the MP2, 2b:Mb and 3b:Mb methods.

Mode	MP2	2b:Mb	3b:Mb
1	24	23	23
2	43	41	41
3	62	60	61
4	66	63	64
5	178	178	177
6	188	185	185
7	199	195	196
8	224	222	222
9	234	230	230
10	240	236	236
11	262	259	260
12	291	288	288
13	298	293	293
14	302	296	296
15	416	404	405
16	433	421	423
17	453	442	443
18	465	454	455
19	522	509	510
20	716	697	699
21	787	766	768
22	856	835	836
23	876	854	855
24	974	951	954
25	1650	1669	1669
26	1660	1678	1678
27	1670	1687	1687
28	1688	1704	1704
29	1696	1711	1711
30	3359	3428	3424
31	3441	3501	3496
32	3451	3509	3505
33	3497	3549	3545
34	3506	3556	3553
35	3898	3879	3880
36	3900	3881	3882
37	3902	3882	3883
38	3903	3883	3884
39	3904	3885	3886

TABLE S34. haTZ harmonic vibrational frequencies (ω in cm^{-1}) for the optimized C_1 $Face_2$ $Ar(H_2O)_5$ complex using the MP2, 2b:Mb and 3b:Mb methods.

Mode	MP2	2b:Mb	3b:Mb
1	23	24	23
2	35	34	33
3	42	43	41
4	44	44	43
5	50	49	49
6	69	67	66
7	71	69	69
8	179	178	178
9	193	189	188
10	207	202	201
11	230	226	226
12	233	229	229
13	244	239	239
14	268	265	265
15	296	292	292
16	299	294	294
17	303	297	297
18	422	409	410
19	435	423	424
20	450	439	440
21	463	451	453
22	533	520	521
23	716	697	698
24	790	769	771
25	852	831	832
26	882	860	861
27	975	952	955
28	1650	1669	1669
29	1660	1677	1677
30	1673	1689	1688
31	1689	1704	1704
32	1698	1713	1712
33	3352	3423	3419
34	3434	3495	3490
35	3447	3505	3501
36	3492	3545	3541
37	3502	3552	3550
38	3896	3877	3878
39	3896	3877	3879
40	3898	3880	3881
41	3899	3881	3882
42	3900	3882	3883

TABLE S35. haTZ harmonic vibrational frequencies (ω in cm^{-1}) for the optimized C_1 Face$_3$ $Ar(H_2O)_5$ complex using the MP2, 2b:Mb and 3b:Mb methods.

Mode	MP2	2b:Mb	3b:Mb
1	20	21	20
2	31	32	31
3	37	37	37
4	42	43	41
5	50	49	49
6	68	66	66
7	71	70	70
8	178	178	177
9	192	189	189
10	202	199	198
11	225	223	223
12	234	231	230
13	240	237	237
14	267	264	264
15	296	292	292
16	300	295	295
17	303	298	297
18	418	406	407
19	436	424	425
20	456	445	446
21	461	449	450
22	521	509	510
23	719	700	701
24	793	772	774
25	861	839	841
26	875	853	855
27	974	951	954
28	1650	1669	1669
29	1661	1679	1678
30	1670	1687	1686
31	1689	1704	1704
32	1698	1712	1712
33	3353	3423	3419
34	3436	3497	3492
35	3445	3504	3500
36	3492	3545	3541
37	3503	3553	3550
38	3894	3876	3877
39	3896	3878	3879
40	3899	3879	3881
41	3900	3880	3882
42	3903	3883	3884

TABLE S36. haTZ harmonic vibrational frequencies (ω in cm^{-1}) for the optimized C_1 Edge $Ar(H_2O)_5$ complex using the MP2, 2b:Mb and 3b:Mb methods.

Mode	MP2	2b:Mb	3b:Mb
1	9	9	9
2	23	23	23
3	25	25	25
4	37	37	37
5	43	42	42
6	64	62	63
7	67	65	66
8	179	178	178
9	186	184	184
10	202	198	198
11	226	224	224
12	234	231	231
13	241	238	238
14	262	260	260
15	291	289	289
16	299	294	294
17	302	296	297
18	417	405	406
19	432	421	422
20	453	442	443
21	465	454	455
22	526	513	515
23	717	698	700
24	787	766	769
25	856	835	837
26	878	856	858
27	975	953	955
28	1650	1669	1669
29	1660	1678	1677
30	1671	1687	1687
31	1688	1703	1703
32	1697	1711	1711
33	3358	3427	3422
34	3440	3500	3495
35	3449	3507	3503
36	3496	3548	3544
37	3503	3554	3550
38	3898	3879	3880
39	3900	3881	3881
40	3901	3882	3883
41	3901	3882	3883
42	3902	3883	3884

III. COUNTERPOISE-CORRECTED BINDING AND INTERACTION ENERGIES

TABLE S37. MP2/haTZ electronic and counterpoise-corrected binding energies (E_{bind} and E_{bind}^{CP}, respectively) and interaction energies (E_{int} and E_{int}^{CP}, respectively) in kJ mol^{-1} for the MP2/haTZ optimized $Ar(H_2O)_3$ complexes.

Label	E_{bind}	E_{bind}^{CP}	E_{int}	E_{int}^{CP}
C_1 Face$_1$	−3.88	−2.80	−3.90	−2.82
C_1 Face$_2$	−3.52	−2.55	−3.53	−2.54
C_1 Edge	−2.54	−1.80	−2.54	−1.81
C_3 Face$_0$	−4.02	−2.89	−4.03	−2.89
C_3 Face$_3$	−2.95	−2.09	−2.95	−2.07

TABLE S38. MP2/haTZ electronic and counterpoise-corrected binding energies (E_{bind} and E_{bind}^{CP}, respectively) and interaction energies (E_{int} and E_{int}^{CP}, respectively) in kJ mol^{-1} for the MP2/haTZ optimized $Ar(H_2O)_4$ complexes.

Label	E_{bind}	E_{bind}^{CP}	E_{int}	E_{int}^{CP}
C_2 Face	−4.65	−3.39	−4.71	−3.39
C_1 Face	−5.03	−3.68	−5.05	−3.68
C_1 Edge	−2.82	−2.01	−2.83	−2.01
C_4 Face	−5.46	−3.93	−5.48	−3.95

TABLE S39. MP2/haTZ electronic and counterpoise-corrected binding energies (E_{bind} and E_{bind}^{CP}, respectively) and interaction energies (E_{int} and E_{int}^{CP}, respectively)in kJ mol^{-1} for the MP2/haTZ optimized $Ar(H_2O)_5$ complexes.

Label	E_{bind}	E_{bind}^{CP}	E_{int}	E_{int}^{CP}
C_1 Face$_2$	−5.85	−4.39	−5.97	−4.36
C_1 Face$_3$	−5.36	−4.02	−5.50	−3.99
C_1 Edge	−2.95	−2.13	−2.96	−2.09
